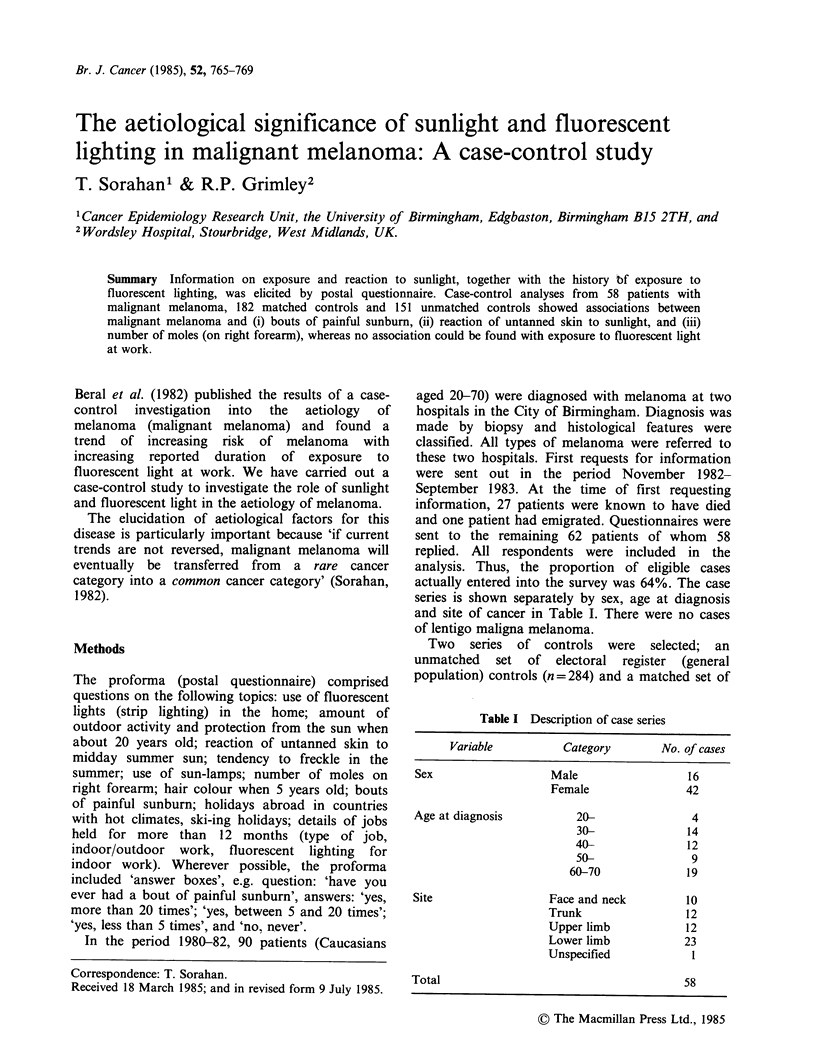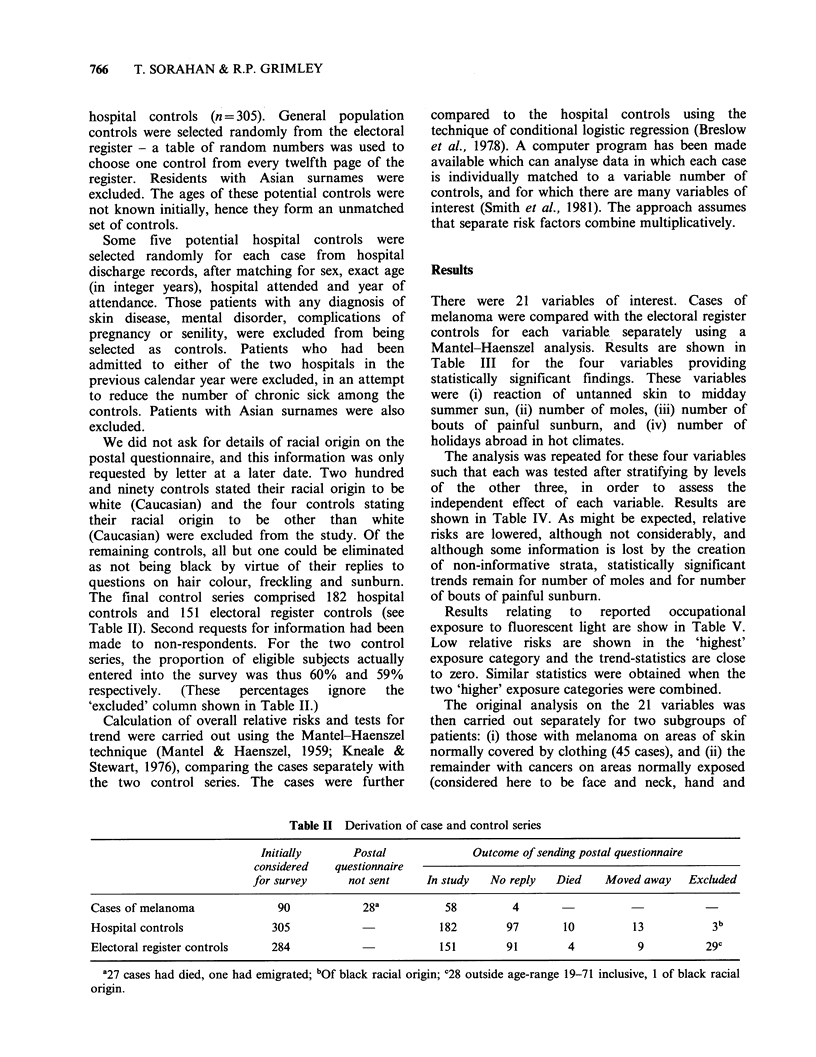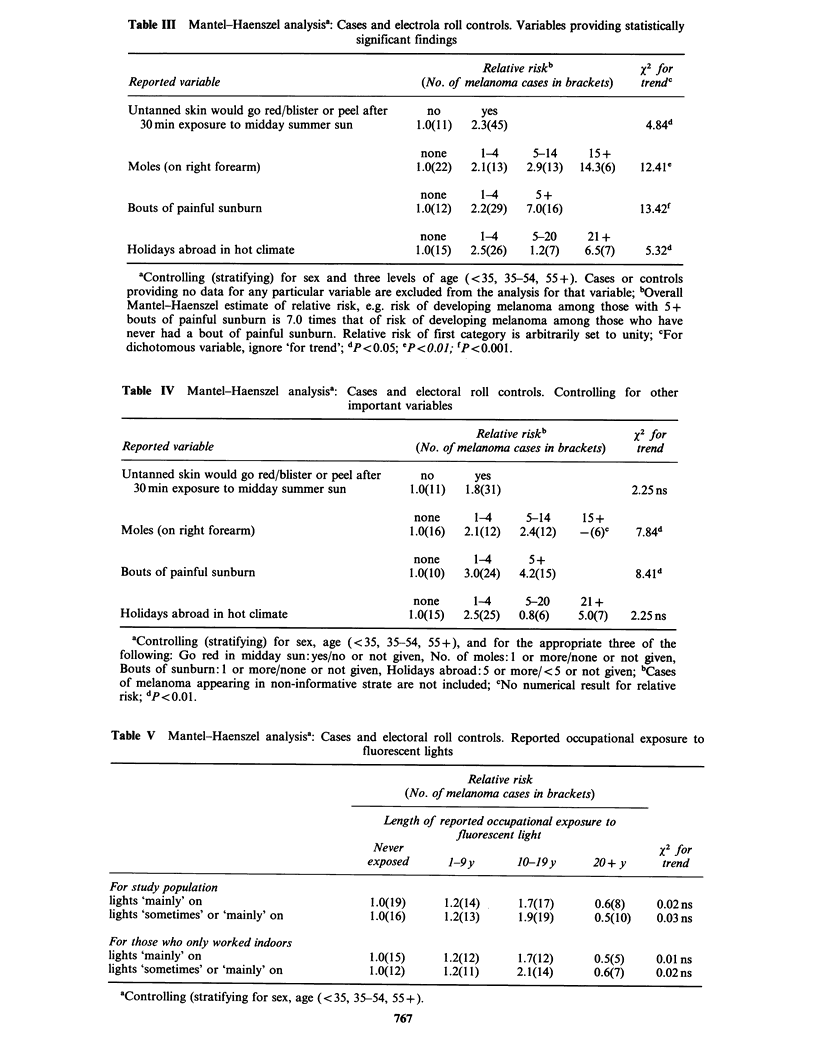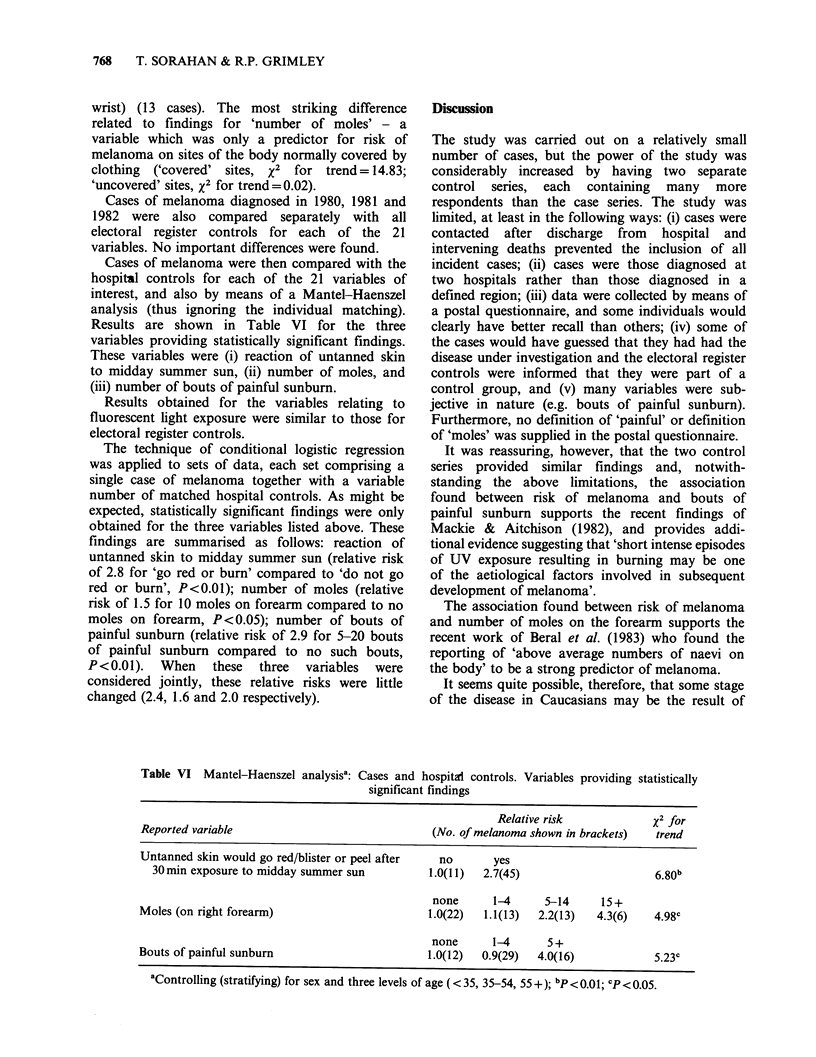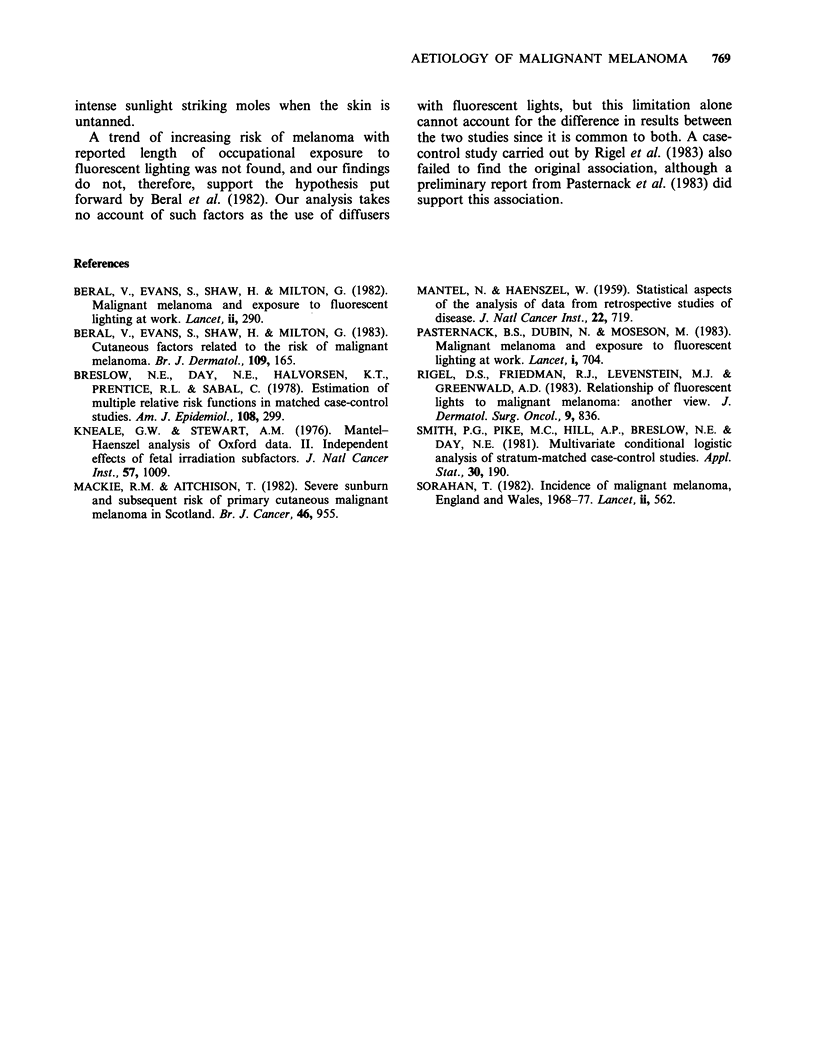# The aetiological significance of sunlight and fluorescent lighting in malignant melanoma: a case-control study.

**DOI:** 10.1038/bjc.1985.255

**Published:** 1985-11

**Authors:** T. Sorahan, R. P. Grimley

## Abstract

Information on exposure and reaction to sunlight, together with the history of exposure to fluorescent lighting, was elicited by postal questionnaire. Case-control analysis from 58 patients with malignant melanoma, 182 matched controls and 151 unmatched controls showed associations between malignant melanoma and (i) bouts of painful sunburn, (ii) reaction of untanned skin to sunlight, and (iii) number of moles (on right forearm), whereas no association could be found with exposure to fluorescent light at work.


Br. J. Cancer (1985), 52, 765–769

### The aetiological significance of sunlight and fluorescent lighting in malignant melanoma: A case-control study

T. Sorahan[1] & R.P. Grimley[2]

[1]*Cancer Epidemiology Research Unit, the University of Birmingham, Edgbaston, Birmingham B15 2TH, and*
[2]*Wordsley Hospital, Stourbridge, West Midlands, UK.*

**Summary** Information on exposure and reaction to sunlight, together with the history of exposure to fluorescent lighting, was elicited by postal questionnaire. Case-control analyses from 58 patients with malignant melanoma, 182 matched controls and 151 unmatched controls showed associations between malignant melanoma and (i) bouts of painful sunburn, (ii) reaction of untanned skin to sunlight, and (iii) number of moles (on right forearm), whereas no association could be found with exposure to fluorescent light at work.

Beral *et al.* (1982) published the results of a case-control investigation into the aetiology of melanoma (malignant melanoma) and found a trend of increasing risk of melanoma with increasing reported duration of exposure to fluorescent light at work. We have carried out a case-control study to investigate the role of sunlight and fluorescent light in the aetiology of melanoma.

The elucidation of aetiological factors for this disease is particularly important because 'if current trends are not reversed, malignant melanoma will eventually be transferred from a *rare* cancer category into a *common* cancer category' (Sorahan, 1982).

### Methods

The proforma (postal questionnaire) comprised questions on the following topics: use of fluorescent lights (strip lighting) in the home; amount of outdoor activity and protection from the sun when about 20 years old; reaction of untanned skin to midday summer sun; tendency to freckle in the summer; use of sun-lamps; number of moles on right forearm; hair colour when 5 years old; bouts of painful sunburn; holidays abroad in countries with hot climates, ski-ing holidays; details of jobs held for more than 12 months (type of job, indoor/outdoor work, fluorescent lighting for indoor work). Wherever possible, the proforma included 'answer boxes', e.g. question: 'have you ever had a bout of painful sunburn', answers: 'yes, more than 20 times'; 'yes, between 5 and 20 times'; 'yes, less than 5 times', and 'no, never'.

In the period 1980–82, 90 patients (Caucasians aged 20–70) were diagnosed with melanoma at two hospitals in the City of Birmingham. Diagnosis was made by biopsy and histological features were classified. All types of melanoma were referred to these two hospitals. First requests for information were sent out in the period November 1982–September 1983. At the time of first requesting information, 27 patients were known to have died and one patient had emigrated. Questionnaires were sent to the remaining 62 patients of whom 58 replied. All respondents were included in the analysis. Thus, the proportion of eligible cases actually entered into the survey was 64%. The case series is shown separately by sex, age at diagnosis and site of cancer in Table I. There were no cases of lentigo maligna melanoma.

Two series of controls were selected; an unmatched set of electoral register (general population) controls ($n=284$) and a matched set of

**Table I** Description of case series

| *Variable* | *Category* | *No. of cases* |
|---|---|---|
| Sex | Male | 16 |
| | Female | 42 |
| Age at diagnosis | 20– | 4 |
| | 30– | 14 |
| | 40– | 12 |
| | 50– | 9 |
| | 60–70 | 19 |
| Site | Face and neck | 10 |
| | Trunk | 12 |
| | Upper limb | 12 |
| | Lower limb | 23 |
| | Unspecified | 1 |
| Total | | 58 |

Correspondence: T. Sorahan.
Received 18 March 1985; and in revised form 9 July 1985.

© The Macmillan Press Ltd., 1985

hospital controls ($n = 305$). General population controls were selected randomly from the electoral register – a table of random numbers was used to choose one control from every twelfth page of the register. Residents with Asian surnames were excluded. The ages of these potential controls were not known initially, hence they form an unmatched set of controls.

Some five potential hospital controls were selected randomly for each case from hospital discharge records, after matching for sex, exact age (in integer years), hospital attended and year of attendance. Those patients with any diagnosis of skin disease, mental disorder, complications of pregnancy or senility, were excluded from being selected as controls. Patients who had been admitted to either of the two hospitals in the previous calendar year were excluded, in an attempt to reduce the number of chronic sick among the controls. Patients with Asian surnames were also excluded.

We did not ask for details of racial origin on the postal questionnaire, and this information was only requested by letter at a later date. Two hundred and ninety controls stated their racial origin to be white (Caucasian) and the four controls stating their racial origin to be other than white (Caucasian) were excluded from the study. Of the remaining controls, all but one could be eliminated as not being black by virtue of their replies to questions on hair colour, freckling and sunburn. The final control series comprised 182 hospital controls and 151 electoral register controls (see Table II). Second requests for information had been made to non-respondents. For the two control series, the proportion of eligible subjects actually entered into the survey was thus 60% and 59% respectively. (These percentages ignore the 'excluded' column shown in Table II.)

Calculation of overall relative risks and tests for trend were carried out using the Mantel–Haenszel technique (Mantel & Haenszel, 1959; Kneale & Stewart, 1976), comparing the cases separately with the two control series. The cases were further compared to the hospital controls using the technique of conditional logistic regression (Breslow *et al.*, 1978). A computer program has been made available which can analyse data in which each case is individually matched to a variable number of controls, and for which there are many variables of interest (Smith *et al.*, 1981). The approach assumes that separate risk factors combine multiplicatively.

### Results

There were 21 variables of interest. Cases of melanoma were compared with the electoral register controls for each variable separately using a Mantel–Haenszel analysis. Results are shown in Table III for the four variables providing statistically significant findings. These variables were (i) reaction of untanned skin to midday summer sun, (ii) number of moles, (iii) number of bouts of painful sunburn, and (iv) number of holidays abroad in hot climates.

The analysis was repeated for these four variables such that each was tested after stratifying by levels of the other three, in order to assess the independent effect of each variable. Results are shown in Table IV. As might be expected, relative risks are lowered, although not considerably, and although some information is lost by the creation of non-informative strata, statistically significant trends remain for number of moles and for number of bouts of painful sunburn.

Results relating to reported occupational exposure to fluorescent light are show in Table V. Low relative risks are shown in the 'highest' exposure category and the trend-statistics are close to zero. Similar statistics were obtained when the two 'higher' exposure categories were combined.

The original analysis on the 21 variables was then carried out separately for two subgroups of patients: (i) those with melanoma on areas of skin normally covered by clothing (45 cases), and (ii) the remainder with cancers on areas normally exposed (considered here to be face and neck, hand and

**Table II** Derivation of case and control series

| | *Initially considered for survey* | *Postal questionnaire not sent* | *Outcome of sending postal questionnaire* | | | | |
|---|---|---|---|---|---|---|---|
| | | | *In study* | *No reply* | *Died* | *Moved away* | *Excluded* |
| Cases of melanoma | 90 | 28[a] | 58 | 4 | — | — | — |
| Hospital controls | 305 | — | 182 | 97 | 10 | 13 | 3[b] |
| Electoral register controls | 284 | — | 151 | 91 | 4 | 9 | 29[c] |

[a]27 cases had died, one had emigrated; [b]Of black racial origin; [c]28 outside age-range 19–71 inclusive, 1 of black racial origin.

**Table III** Mantel–Haenszel analysis[a]: Cases and electrola roll controls. Variables providing statistically significant findings

| *Reported variable* | *Relative risk[b] (No. of melanoma cases in brackets)* | | | | $\chi^2$ *for trend*[c] |
|---|---|---|---|---|---|
| Untanned skin would go red/blister or peel after 30 min exposure to midday summer sun | no<br>1.0(11) | yes<br>2.3(45) | | | 4.84[d] |
| Moles (on right forearm) | none<br>1.0(22) | 1–4<br>2.1(13) | 5–14<br>2.9(13) | 15+<br>14.3(6) | 12.41[e] |
| Bouts of painful sunburn | none<br>1.0(12) | 1–4<br>2.2(29) | 5+<br>7.0(16) | | 13.42[f] |
| Holidays abroad in hot climate | none<br>1.0(15) | 1–4<br>2.5(26) | 5–20<br>1.2(7) | 21+<br>6.5(7) | 5.32[d] |

[a]Controlling (stratifying) for sex and three levels of age (<35, 35–54, 55+). Cases or controls providing no data for any particular variable are excluded from the analysis for that variable; [b]Overall Mantel–Haenszel estimate of relative risk, e.g. risk of developing melanoma among those with 5+ bouts of painful sunburn is 7.0 times that of risk of developing melanoma among those who have never had a bout of painful sunburn. Relative risk of first category is arbitrarily set to unity; [c]For dichotomous variable, ignore 'for trend'; [d]$P<0.05$; [e]$P<0.01$; [f]$P<0.001$.

**Table IV** Mantel–Haenszel analysis[a]: Cases and electoral roll controls. Controlling for other important variables

| *Reported variable* | *Relative risk[b] (No. of melanoma cases in brackets)* | | | | $\chi^2$ *for trend* |
|---|---|---|---|---|---|
| Untanned skin would go red/blister or peel after 30 min exposure to midday summer sun | no<br>1.0(11) | yes<br>1.8(31) | | | 2.25 ns |
| Moles (on right forearm) | none<br>1.0(16) | 1–4<br>2.1(12) | 5–14<br>2.4(12) | 15+<br>–(6)[c] | 7.84[d] |
| Bouts of painful sunburn | none<br>1.0(10) | 1–4<br>3.0(24) | 5+<br>4.2(15) | | 8.41[d] |
| Holidays abroad in hot climate | none<br>1.0(15) | 1–4<br>2.5(25) | 5–20<br>0.8(6) | 21+<br>5.0(7) | 2.25 ns |

[a]Controlling (stratifying) for sex, age (<35, 35–54, 55+), and for the appropriate three of the following: Go red in midday sun: yes/no or not given, No. of moles: 1 or more/none or not given, Bouts of sunburn: 1 or more/none or not given, Holidays abroad: 5 or more/<5 or not given; [b]Cases of melanoma appearing in non-informative strate are not included; [c]No numerical result for relative risk; [d]$P<0.01$.

**Table V** Mantel–Haenszel analysis[a]: Cases and electoral roll controls. Reported occupational exposure to fluorescent lights

| | *Relative risk (No. of melanoma cases in brackets)* | | | | |
|---|---|---|---|---|---|
| | *Length of reported occupational exposure to fluorescent light* | | | | |
| | *Never exposed* | *1–9 y* | *10–19 y* | *20+ y* | $\chi^2$ *for trend* |
| *For study population* | | | | | |
| lights 'mainly' on | 1.0(19) | 1.2(14) | 1.7(17) | 0.6(8) | 0.02 ns |
| lights 'sometimes' or 'mainly' on | 1.0(16) | 1.2(13) | 1.9(19) | 0.5(10) | 0.03 ns |
| *For those who only worked indoors* | | | | | |
| lights 'mainly' on | 1.0(15) | 1.2(12) | 1.7(12) | 0.5(5) | 0.01 ns |
| lights 'sometimes' or 'mainly' on | 1.0(12) | 1.2(11) | 2.1(14) | 0.6(7) | 0.02 ns |

[a]Controlling (stratifying for sex, age (<35, 35–54, 55+).

wrist) (13 cases). The most striking difference related to findings for 'number of moles' – a variable which was only a predictor for risk of melanoma on sites of the body normally covered by clothing ('covered' sites, $\chi^2$ for trend = 14.83; 'uncovered' sites, $\chi^2$ for trend = 0.02).

Cases of melanoma diagnosed in 1980, 1981 and 1982 were also compared separately with all electoral register controls for each of the 21 variables. No important differences were found.

Cases of melanoma were then compared with the hospital controls for each of the 21 variables of interest, and also by means of a Mantel–Haenszel analysis (thus ignoring the individual matching). Results are shown in Table VI for the three variables providing statistically significant findings. These variables were (i) reaction of untanned skin to midday summer sun, (ii) number of moles, and (iii) number of bouts of painful sunburn.

Results obtained for the variables relating to fluorescent light exposure were similar to those for electoral register controls.

The technique of conditional logistic regression was applied to sets of data, each set comprising a single case of melanoma together with a variable number of matched hospital controls. As might be expected, statistically significant findings were only obtained for the three variables listed above. These findings are summarised as follows: reaction of untanned skin to midday summer sun (relative risk of 2.8 for 'go red or burn' compared to 'do not go red or burn', $P<0.01$); number of moles (relative risk of 1.5 for 10 moles on forearm compared to no moles on forearm, $P<0.05$); number of bouts of painful sunburn (relative risk of 2.9 for 5–20 bouts of painful sunburn compared to no such bouts, $P<0.01$). When these three variables were considered jointly, these relative risks were little changed (2.4, 1.6 and 2.0 respectively).

### Discussion

The study was carried out on a relatively small number of cases, but the power of the study was considerably increased by having two separate control series, each containing many more respondents than the case series. The study was limited, at least in the following ways: (i) cases were contacted after discharge from hospital and intervening deaths prevented the inclusion of all incident cases; (ii) cases were those diagnosed at two hospitals rather than those diagnosed in a defined region; (iii) data were collected by means of a postal questionnaire, and some individuals would clearly have better recall than others; (iv) some of the cases would have guessed that they had had the disease under investigation and the electoral register controls were informed that they were part of a control group, and (v) many variables were subjective in nature (e.g. bouts of painful sunburn). Furthermore, no definition of 'painful' or definition of 'moles' was supplied in the postal questionnaire.

It was reassuring, however, that the two control series provided similar findings and, notwithstanding the above limitations, the association found between risk of melanoma and bouts of painful sunburn supports the recent findings of Mackie & Aitchison (1982), and provides additional evidence suggesting that 'short intense episodes of UV exposure resulting in burning may be one of the aetiological factors involved in subsequent development of melanoma'.

The association found between risk of melanoma and number of moles on the forearm supports the recent work of Beral *et al.* (1983) who found the reporting of 'above average numbers of naevi on the body' to be a strong predictor of melanoma.

It seems quite possible, therefore, that some stage of the disease in Caucasians may be the result of

**Table VI** Mantel–Haenszel analysis[a]: Cases and hospital controls. Variables providing statistically significant findings

| *Reported variable* | *Relative risk (No. of melanoma shown in brackets)* | | | | *$\chi^2$ for trend* |
|---|---|---|---|---|---|
| Untanned skin would go red/blister or peel after 30 min exposure to midday summer sun | no<br>1.0(11) | yes<br>2.7(45) | | | 6.80[b] |
| Moles (on right forearm) | none<br>1.0(22) | 1–4<br>1.1(13) | 5–14<br>2.2(13) | 15+<br>4.3(6) | 4.98[c] |
| Bouts of painful sunburn | none<br>1.0(12) | 1–4<br>0.9(29) | 5+<br>4.0(16) | | 5.23[c] |

[a]Controlling (stratifying) for sex and three levels of age (<35, 35–54, 55+); [b]$P<0.01$; [c]$P<0.05$.

intense sunlight striking moles when the skin is untanned.

A trend of increasing risk of melanoma with reported length of occupational exposure to fluorescent lighting was not found, and our findings do not, therefore, support the hypothesis put forward by Beral *et al.* (1982). Our analysis takes no account of such factors as the use of diffusers with fluorescent lights, but this limitation alone cannot account for the difference in results between the two studies since it is common to both. A case-control study carried out by Rigel *et al.* (1983) also failed to find the original association, although a preliminary report from Pasternack *et al.* (1983) did support this association.

### References

BERAL, V., EVANS, S., SHAW, H. & MILTON, G. (1982). Malignant melanoma and exposure to fluorescent lighting at work. *Lancet*, **ii**, 290.

BERAL, V., EVANS, S., SHAW, H. & MILTON, G. (1983). Cutaneous factors related to the risk of malignant melanoma. *Br. J. Dermatol.*, **109**, 165.

BRESLOW, N.E., DAY, N.E., HALVORSEN, K.T., PRENTICE, R.L. & SABAL, C. (1978). Estimation of multiple relative risk functions in matched case-control studies. *Am. J. Epidemiol.*, **108**, 299.

KNEALE, G.W. & STEWART, A.M. (1976). Mantel–Haenszel analysis of Oxford data. II. Independent effects of fetal irradiation subfactors. *J. Natl Cancer Inst.*, **57**, 1009.

MACKIE, R.M. & AITCHISON, T. (1982). Severe sunburn and subsequent risk of primary cutaneous malignant melanoma in Scotland. *Br. J. Cancer*, **46**, 955.

MANTEL, N. & HAENSZEL, W. (1959). Statistical aspects of the analysis of data from retrospective studies of disease. *J. Natl Cancer Inst.*, **22**, 719.

PASTERNACK, B.S., DUBIN, N. & MOSESON, M. (1983). Malignant melanoma and exposure to fluorescent lighting at work. *Lancet*, **i**, 704.

RIGEL, D.S., FRIEDMAN, R.J., LEVENSTEIN, M.J. & GREENWALD, A.D. (1983). Relationship of fluorescent lights to malignant melanoma: another view. *J. Dermatol. Surg. Oncol.*, **9**, 836.

SMITH, P.G., PIKE, M.C., HILL, A.P., BRESLOW, N.E. & DAY, N.E. (1981). Multivariate conditional logistic analysis of stratum-matched case-control studies. *Appl. Stat.*, **30**, 190.

SORAHAN, T. (1982). Incidence of malignant melanoma, England and Wales, 1968–77. *Lancet*, **ii**, 562.